# Effect of Solution Treatment Temperature on Microstructural Evolution and Mechanical Properties of GH4698 Superalloy

**DOI:** 10.3390/ma19091806

**Published:** 2026-04-29

**Authors:** Xiaofeng Yan, Jianxin Dong, He Jiang

**Affiliations:** 1 High-Temperature Materials Research Laboratory, School of Materials Science and Engineering, University of Science and Technology Beijing, Beijing 100083, China; gaona427@163.com (X.Y.); jxdong@ustb.edu.cn (J.D.); 2 Institute of High-Temperature Materials, Central Iron & Steel Research Institute, Beijing 100081, China

**Keywords:** superalloy, GH4698, microstructure, deformation mechanisms, tensile behavior

## Abstract

This study systematically investigates the effects of solution temperature ranging from 1060 to 1150 °C on grain growth kinetics, microstructural evolution, and tensile properties of GH4698 superalloys. The results indicate that grain size coarsens parabolically with increasing solution temperature. Based on the Sellars model, the grain growth time exponent n is determined to be 3.4 and the activation energy Q is 478.7 kJ·mol^−1^. This confirms that the grain growth process is significantly influenced by both MC carbide pinning and alloying element drag effects. Additionally, due to the coarsening of grains, the precipitation density of M_23_C_6_ carbides per unit grain boundary length increased from 0.26 μm^−1^ to 0.39 μm^−1^. The ultimate tensile strength at room temperature decreased from 1268 MPa to 1226 MPa, and the yield strength decreased from 840 MPa to 807 MPa, while the elongation remained at 28–32%. At 700 °C, the ultimate tensile strength decreases from 974 MPa to 904 MPa, and the yield strength decreases from 755 MPa to 696 MPa, with the elongation remaining at ~6%. Quantitative analysis reveals that the decrease in strength is primarily due to the weakening of grain boundary strengthening caused by grain coarsening. At 700 °C, the deformation mechanism transitions from dislocation shearing at room temperature to stacking fault shearing. This not only leads to a reduction in strength but also, accompanied by grain boundary weakening, results in a decrease in elongation.

## 1. Introduction

Nickel-based superalloys, due to the excellent high-temperature strength, oxidation resistance, and creep resistance, have become an irreplaceable key material for hot-section components in aircraft engines and gas turbines [1,2]. Among them, GH4698 superalloy, as a typical precipitation-strengthened Ni-based superalloy, achieves high-temperature strengthening through L1_2_-ordered γ′ phase coherent precipitation, and is widely used in the manufacture of hot-end components such as turbine disks and fasteners operating at temperatures below 750 °C. These components are exposed to high centrifugal stresses and complex thermomechanical fatigue loads under extreme operating conditions, and the service reliability and lifespan of these components are closely related to the microstructure of the alloy, particularly grain size, the size/distribution of the γ′ phase, and the morphology and stability of carbides. Precise control of the microstructure through thermomechanical processing and subsequent heat treatment is the essential approach to optimize the overall properties. The present research on GH4698 alloy primarily focuses on optimizing hot deformation processes [3,4], investigating abnormal grain growth behavior during solution treatment [5], and enhancing tensile properties and microstructural stability by regulating trace elements and examining long-term aging effects [6,7]. Although these works provide an important foundation for understanding the relationship between processing, microstructure, and properties, there is still a relative lack of research on the role of solution treatment, as the critical link connecting hot working and final aging strengthening, in systematically controlling the microstructure of GH4698 superalloy and thereby determining its mechanical properties.

Extensive studies have reported on the optimization of alloy microstructure and properties through controlled solution treatment. Shi et al. [8] pointed out that the dissolution of MC carbides during solution treatment releases alloying elements, promoting the preferential nucleation and rapid growth of the γ′ phase, thereby forming a coarsened γ′ phase. Gai et al. [9] found that solution treatment at 1130 °C in GH4151 superalloy formed medium-sized, near-spherical γ′ secondary phases with a high-volume fraction, whereas treatment at 1140 °C resulted in microstructural deterioration due to excessive coarsening of the γ′ phase. Chen et al. [10] demonstrated that as the temperature increased (1185 °C → 1260 °C → 1300 °C), the K447A superalloy successively underwent grain recovery, recrystallization, and accelerated grain growth. A uniform cubic γ′ phase was obtained after treatment at 1260 °C. Moreover, Zhong et al. [11] indicated that increasing the solution temperature promotes grain growth, continuous distribution of carbides along grain boundaries, and a gradual transition of the γ′ phase from a bimodal distribution to a unimodal distribution. Joseph et al. [12] demonstrated that regulating the γ′ phase size and grain boundary carbide morphology in Haynes 282 alloy through solution treatment temperature enables synergistic matching of strength and toughness. Wan et al. [13] found that an increase in solution treatment temperature led to grain coarsening and secondary γ′ phase growth in the U720Li alloy. This resulted in reduced strength and ductility at room and intermediate temperatures. Zhong et al. [11,14] further reported that as the solution temperature increases, the creep life of the alloy first increases and then decreases, achieving optimal comprehensive properties at 1000 °C. Additionally, the increase in solution temperature leads to a decrease in low-cycle fatigue life. Atabay et al. [15] showed that in Rene 41 superalloy, sub-solution treatment below the γ′ phase dissolution temperature promotes the formation of fine, uniformly distributed γ′ phases. This facilitates an increase in elongation while maintaining high strength.

Although previous studies have revealed general principles governing the microstructure and properties of Ni-based superalloys through solution treatment, quantitative structure property relationships between solution temperature, microstructure, and tensile properties remain scarce for GH4698 superalloy. For instance, the quantitative relationship between solution temperature and grain size has not been established, and key kinetic parameters, such as the grain growth activation energy (Q) and grain growth exponent (*n*), remain unreported, limiting the quantitative prediction of grain evolution. Moreover, the quantitative variation in mechanical properties with solution temperature remains unclear, and the contributions of various strengthening mechanisms to yield strength have not been systematically deconvoluted. Therefore, this work quantitatively established the grain growth kinetic model of GH4698 using the Sellars model, obtaining the grain growth exponent (*n*) and apparent activation energy (Q). The intrinsic correlations among solution temperature, microstructure, and tensile properties were established, and the contributions of various strengthening mechanisms to yield strength were quantitatively deconvoluted, elucidating the evolution mechanisms of mechanical properties with solution temperature. These findings provide a theoretical basis and data support for optimizing the heat treatment process of the GH4698 alloy.

## 2. Materials and Methods

### 2.1. Materials Preparation

The GH4698 superalloy used in the experiment was produced via a dual-process combination of vacuum induction melting (VIM) and electroslag remelting (ESR) to obtain master alloy ingots with precise composition and low gas content. The actual composition was determined by inductively coupled plasma optical emission spectrometry (ICP-OES) and was presented in Table 1. After homogenization treatment, the ingot was forged at 1160 °C into rods with a diameter of 16 mm. To investigate the effect of solution temperature on the grain growth kinetics and short-term tensile behavior of the GH4698 superalloy, specimens with dimensions of 70 mm were cut from the rods using wire electrical discharge machining.

**Figure 1:**
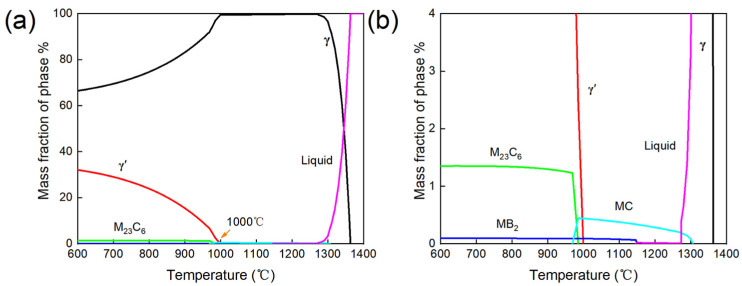
Thermodynamic equilibrium phase diagram of the GH4698 superalloy: (**a**) overall view, (**b**) enlarged view.

To investigate the grain growth kinetics of GH4698 superalloy, the equilibrium phase diagram of GH4698 superalloy was calculated using JMatPro 7.0 software, with the results shown in Figure 1. The calculations indicate that the equilibrium microstructure of the alloy primarily consists of the γ matrix, γ′ phase, MC carbides, M_23_C_6_ carbides, and borides, with the complete dissolution temperature of the γ′ phase being approximately 1000 °C. Furthermore, the thermodynamic calculations reveal that, under equilibrium conditions, MC carbides tend to transform into M_23_C_6_ carbides. Based on the above phase diagram analysis, solution treatment temperatures of 1060 °C, 1080 °C, 1090 °C, 1100 °C, 1120 °C, and 1150 °C were selected for this study. These temperatures are all above the complete dissolution temperature of the γ′ phase, thereby eliminating the interference of γ′ phase on grain growth. The holding times were set to 2, 4, 6, and 8 h, respectively. After solution treatment, the specimens held for 8 h were further processed with a two-step aging treatment, which consisted of holding at 1000 °C for 4 h followed by air cooling, and then at 775 °C for 16 h followed by air cooling. This aging treatment was designed to obtain a typical γ′ phase distribution for subsequent short-term mechanical property testing. Detailed heat treatment information and sample designations are presented in Table 2.

The heat-treated bars were cut into standard tensile specimens with a diameter of 12 mm and a gauge length of 55 mm. Quasi-static tensile tests were conducted according to the GB/T 228.1-2021 and GB/T 228.2-2015 test standards [16,17]. A segmented control method was applied for the tensile rate: before yielding, the strain rate was maintained at 7 × 10^−5^ s^−1^, while after yielding, the crosshead speed was kept constant at 2.52 mm/min. To ensure the reproducibility of the mechanical properties, three tests were performed under identical conditions, and the average value was taken as the final mechanical property.

### 2.2. Microstructure Examination

The test specimens were sequentially polished using SiC abrasive paper of grit sizes #320, #800, #1500, and #2000, followed by mechanical polishing with a 2.5 μm diamond suspension. A JEOL 7800 field emission scanning electron microscope (Tokyo, Japan) equipped with energy dispersive spectroscopy (EDS) and electron backscatter diffraction (EBSD) was employed to characterize grain size, precipitation phase distribution, and tensile fracture surfaces. The size and volume fraction of precipitates were statistically analyzed using ImagePro Plus 6.0 software. To obtain high-quality EBSD signals, the mechanically polished samples were further subjected to electropolishing in an 80% CH_3_OH + 20% H_2_SO_4_ electrolyte at 15 V for 10 s. EBSD data acquisition was performed at an accelerating voltage of 20 kV, a working distance of 15 mm, and a step size of 2.5 μm. Acquired data were processed and analyzed using Aztec Crystal 2.1 software. The dislocation configuration after tensile deformation was identified using an FEI Tecnai G2 F20 field emission transmission electron microscope (TEM) (Hillsboro, OR, USA) at 200 kV. The TEM sample preparation procedure was as follows: a slice approximately 500 μm thick was first sectioned from the gauge length of the sample along the loading direction, mechanically ground to about 50 μm, and then punched into 3 mm diameter disks. The disks were subsequently thinned by twin-jet electropolishing in a solution of 20% HClO_4_ + 80% CH_3_CH_2_OH at approximately −30 °C, followed by final cleaning using an ion mill under an argon atmosphere.

## 3. Results and Discussion

### 3.1. Microstructure Before Heat Treatment

**Figure 2:**
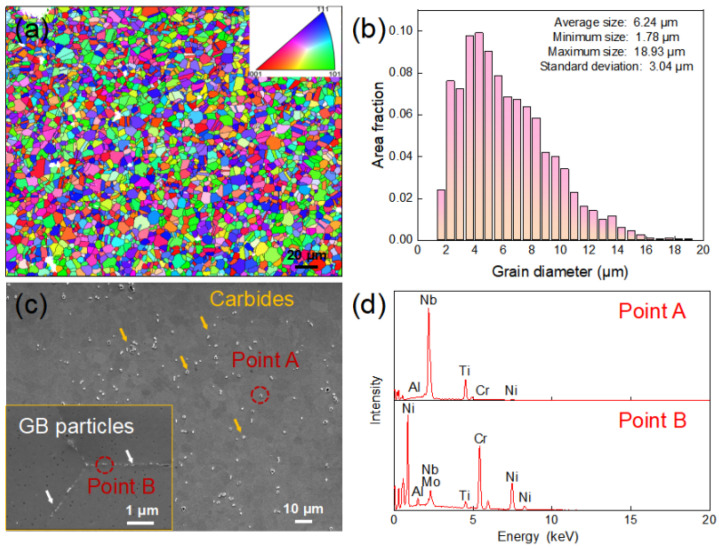
Microstructure of the GH4698 superalloy before heat treatment: (**a**) inverse pole figure, (**b**) Grain size distribution, (**c**) SEM image of carbides, (**d**) EDS analysis in (**c**).

Figure 2 shows the EBSD results of the microstructure of GH4698 superalloy before heat treatment. As illustrated in Figure 1a,b, the alloy matrix consists of uniform and fine equiaxed grains with no significant texture, and the average grain size is approximately 6.24 ± 3.04 μm. Figure 2c presents the corresponding SEM image, revealing a large number of white granular secondary phases dispersed within the matrix. EDS analysis indicates that the white particles in Figure 2b are rich in Nb and Ti, corresponding to (Ni,Ti) C-type MC carbides (Point A in Figure 2d). These carbides mainly form during the later stages of solidification and can effectively pin grain boundaries during heat treatment, reducing grain boundary mobility and thereby refining the grains. Additionally, a small amount of gray carbides were observed distributed along grain boundaries. EDS analysis in Figure 2d revealed that these particles are rich in Cr, corresponding to M_23_C_6_ carbides (Point B).

### 3.2. Temperature Dependence of Grain Size Evolution

**Figure 3:**
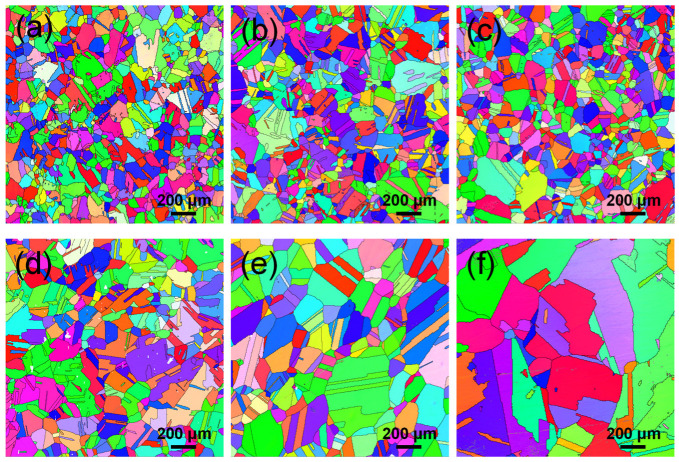
Microstructure of GH4698 superalloy holding at different temperatures for 8 h: (**a**) 1060 °C, (**b**) 1080 °C, (**c**) 1090 °C, (**d**) 1100 °C, (**e**) 1120 °C, (**f**) 1150 °C.

**Figure 4:**
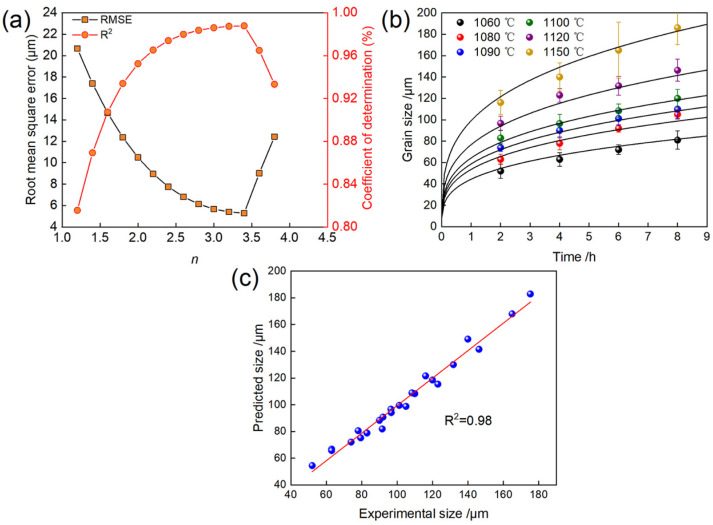
Characterization of grain growth in GH4698 superalloy under different solution treatments: (**a**) Relationship between the time exponent n and the model prediction error, (**b**) kinetic evolution of grain size as a function of holding time, (**c**) comparison between model-predicted and experimentally measured grain sizes.

Figure 3 shows the inverse polar graphs (IPF) of samples subjected to different temperatures under a fixed holding time of 8 h. Quantitative analysis reveals that the temperature is the most significant factor influencing grain growth. As the solution temperature increases from 1060 °C to 1150 °C, the average grain size exhibits a continuous and pronounced growth trend, with specific values of 81.6 μm (1060 °C), 105 μm (1080 °C), 110 μm (1090 °C), 120 μm (1100 °C), 146.3 μm (1120 °C), and 175.3 μm (1150 °C). Detailed grain size data for other holding times (2 h, 4 h, 6 h) are summarized in the scatter plot shown in Figure 4 and the statistical results provided in Table 3, further corroborating the aforementioned temperature dependence. The essence of grain growth is the process of grain boundary migration, and the driving force primarily originates from the reduction in interfacial energy caused by grain boundary curvature. An increase in solution temperature significantly promotes grain growth. On the one hand, grain boundary migration is a thermally activated process; higher temperatures provide greater thermodynamic energy for atoms to cross grain boundaries, thereby markedly enhancing atomic diffusion capacity and accelerating grain boundary migration rates. Secondly, as the solution temperature rises, some fine carbides dissolve or coarsen, weakening their pinning effect on grain boundaries and making it easier for grain boundaries to break free from constraints and migrate. Simultaneously, the segregation of solute atoms at grain boundaries (solute drag effect) tends to diminish at elevated temperatures, further reducing the resistance to grain boundary migration.

Furthermore, at a constant temperature, the grain growth rate is relatively high during the initial 2 h of holding. As the holding time extends to 8 h, the growth trend gradually slows and eventually stabilizes at a steady grain size. This behavior occurs because the high density of grain boundaries in the fine-grained structure at the early stage provides a strong driving force for migration, allowing grain boundaries to move rapidly. As annealing proceeds, the interfacial area decreases significantly, which reduces the driving force for grain boundary migration. Consequently, the growth rate gradually declines and eventually tends to stagnate, leading the microstructure to reach a relatively stable, coarsened state.

To systematically describe the grain growth behavior of the GH4698 superalloy during solution treatment, a complete grain growth kinetic model was established based on the grain evolution within the temperature range of 1060 °C to 1150 °C. According to the Sellars model, the variation in grain size with temperature and time can be expressed as [18]:

(1) $$D^{n}-D_{0}^{n}=At\exp\left(\frac{-Q}{RT}\right)$$

where *t* is the holding time (s), D is the average grain size after solution treatment (μm), D_0_ is the initial grain size (μm), T is the solution temperature (K), *Q* is the activation energy for grain growth (J·mol^−1^), and R is the universal gas constant (8.314 J·mol^−1^·K^−1^), and *n* and *A* are constants. Taking the natural logarithm of Equation (1) gives the linearized form:

(2) $$\ln\left(D^{n}-D_{0}^{n}\right)=\ln A+\ln t-\frac{Q}{RT}$$

To determine the optimal time exponent n for the grain growth kinetics of GH4698 superalloy, the prediction accuracy of the Sellars model corresponding to different *n* values within the range of 1.0 to 4.0 was systematically evaluated. The error analysis results indicate that the root mean square error (RMSE) of the model exhibits a distinct minimum with respect to the variation in *n*. As shown in Figure 4a, when *n* = 3.4, the model achieves the lowest RMSE (5.3 μm) and the highest coefficient of determination, R^2^ (0.940). Therefore, *n* = 3.4 is identified as the optimal time exponent for describing the grain growth of GH4698 superalloy. The grain growth exponent *n* for annealed metals typically ranges from 2 (uniform grain growth process) to 4 (non-uniform or anomalous grain growth process). The value of *n* = 3.4 obtained in this work falls within this reasonable range [19]. Moreover, this value is markedly higher than the theoretical value of *n* = 2, indicating that grain boundary migration in GH4698 superalloy is significantly impeded during solution treatment. This deviation is primarily attributed to the coupled effects of alloying elements and precipitates. Specifically, Mo and Nb, owing to their slower diffusion rates, segregate at grain boundaries to form solute clusters, generating a solute drag effect that effectively reduces the grain boundary migration rate. Additionally, MC carbides distributed within grains and along grain boundaries significantly hinder grain boundary migration through the Zener pinning effect, further suppressing grain growth kinetics [20]. Although localized grain coarsening at high temperatures may contribute to an increased grain growth exponent, no abnormal grain growth-dominated behavior was observed under the present experimental conditions. Therefore, we consider that the obtained *n* = 3.4 authentically reflects the grain growth characteristics of the GH4698 alloy under the combined influence of solute drag and second-phase pinning. The corresponding kinetic parameters *A* = 6.32 × 10^20^ and the activation energy *Q* for grain growth is 478.7 kJ·mol^−1^. The activation energy *Q* is significantly higher than the self-diffusion activation energy of pure Ni (278 kJ·mol^−1^) and the lattice diffusion activation energies of solute elements such as Nb and Mo (200–350 kJ·mol^−1^) [12,21]. It is comparable to reported values for similar Ni-based superalloys like Inconel 718 [22] and Inconel 617 [23] but lower than that of the GH4720Li [24], indicating that its kinetic parameters fall within a reasonable range. Based on this, the Sellars model describing the grain growth kinetics of this alloy can be expressed as:

(3) $$D^{3.4}-D_{0}^{3.4}=6.32\times 10^{20}t\exp\left(\frac{-478,659}{RT}\right)$$

To verify the accuracy of the model, the fitted kinetic equation was used to predict the grain growth behavior of the GH4698 superalloy. The results are shown in Figure 4b. As illustrated, the model predicts that the grain size of the alloy gradually increases with rising solution temperature, while at a given temperature, the grain growth rate decreases with prolonged holding time. These predicted trends are consistent with the experimental observations. Figure 4c further displays the correlation between the predicted and experimentally measured grain sizes. The data points are distributed closely around the y = x reference line, and the coefficient of determination R^2^ reaches 0.98, indicating a strong linear relationship between the predicted and experimental values. In summary, the established grain growth model for the GH4698 superalloy exhibits high predictive accuracy and can be reliably applied to predict the grain size of this alloy within the corresponding processing window.

### 3.3. Effect of Solution Treatment Temperature on Precipitation Phase

**Figure 5:**
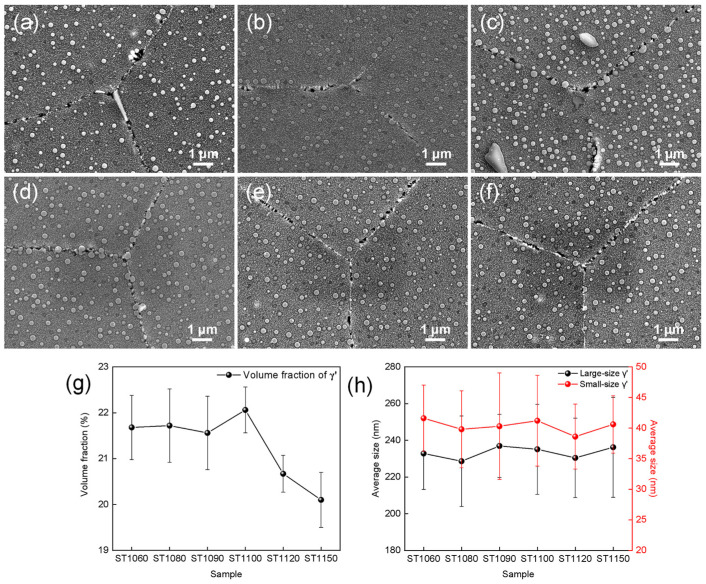
The distribution of γ’ phase in GH4698 superalloy under different heat treatments: (**a**) ST1060, (**b**) ST1080, (**c**) ST1090, (**d**) ST1100, (**e**) ST1120, (**f**) ST1150, (**g**) volume fraction of γ′ phases and (**h**) average size of γ′ phases.

Figure 5 shows the γ′ phase distribution characteristics of GH4698 superalloy after solution treatment at different temperatures followed by a two-step aging process. As can be clearly observed, the γ′ phases are uniformly dispersed throughout the matrix with a typical bimodal size distribution. The size distributions shown in Figure 5a–f and the statistical results in Figure 5h indicate that the larger γ′ phases have an average diameter of approximately 220 nm, while the smaller γ′ phases have an average diameter of approximately 40 nm. Notably, no significant γ’-phase precipitate free regions were observed on either side of grain boundaries under all processing conditions. This indicates that the two-stage aging process effectively promotes uniform precipitation of γ’-phases near grain boundaries, thereby preventing solute atom depletion caused by grain boundary diffusion. Moreover, the statistical results indicate that the overall volume fraction of the γ′ phase after aging remains within the range of 20–22% (Figure 5g). Although the measured values are slightly lower than the equilibrium phase diagram calculations, the deviation is considered reasonable given the inherent errors associated with two-dimensional image statistics. Furthermore, these results further confirm that the precipitation behavior of the γ′ phase is independent of the solution treatment temperature.

**Figure 6:**
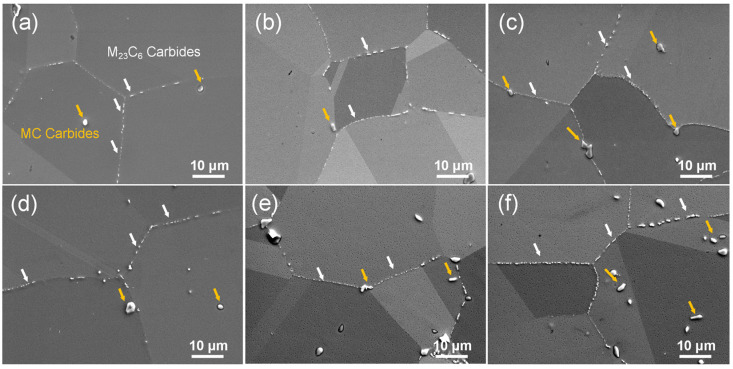
The carbides distribution in GH4698 superalloy under different heat treatments: (**a**) ST1060, (**b**) ST1080, (**c**) ST1090, (**d**) ST1100, (**e**) ST1120, (**f**) ST1150.

Figure 6 illustrates the evolution of carbides after solution treatment at different temperatures followed by a two-step aging process, with the corresponding quantitative statistical results listed in Table 4. Analysis indicates that the (Ti, Nb) C-type carbides remain stable at all solution treatment temperatures, with no significant dissolution or coarsening (indicated by the yellow arrows). Some reports suggest that increasing the solution temperature promotes the decomposition of MC carbides, with the reaction typically following the pattern: MC + γ → M_6_C/M_23_C_6_ + γ′ [10]. As the (W + Mo)/Cr ratio decreases, the decomposition products shift from W/Mo-rich M_6_C to Cr-rich M_23_C_6_. However, the stability of MC carbides observed in the present work differs from the above trend. Qin et al. [25,26] indicated that a higher (Ti + Nb)/(W + Mo) ratio and a higher Nb/Ti ratio can effectively suppress the decomposition of MC carbides. This is primarily because the diffusion rate of Nb is relatively slow, thereby delaying the kinetic process of the decomposition reaction. Zhang et al. [7] also confirmed this behavior during long-term thermal exposure research on GH4698 superalloy, indicating that a higher Nb/Ti ratio is a key factor in maintaining the structural stability of MC carbides at elevated temperatures. Additionally, as the solution temperature increases, the M_23_C_6_ carbides transform from dispersed particles at 1060 °C (Figure 6a) to a nearly continuous distribution along grain boundaries at 1150 °C (Figure 6f). For each specimen, quantitative analysis of grain boundary carbides was performed on five randomly selected SEM images at 2000× magnification. For each image, 3–5 grain boundary segments were randomly chosen for measurement. All carbide particles intersecting the grain boundaries were counted, excluding those indiscernible due to image resolution limitations. The statistical results, presented in Table 4, indicate that the number density of M_23_C_6_ carbides per unit grain boundary length increased significantly with solution temperature, from 0.26 μm^−1^ (ST1060) to 0.38 μm^−1^ (ST1150). This change is attributed to the elevated solution temperature causing significant grain coarsening, reducing the total intergranular area. With a similar total amount of carbide precipitation, the distribution becomes more concentrated in fewer intergranular regions, resulting in a markedly higher carbide density per unit intergranular length.

### 3.4. Effect of Solution Treatment Temperature on the Tensile Properties

**Figure 7:**
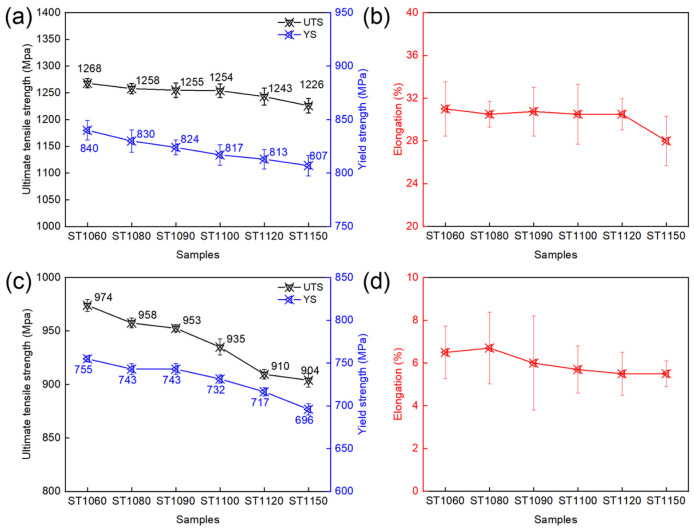
Tensile properties of GH4698 superalloy at (**a**,**b**) RT and (**c**,**d**) 700 °C.

Figure 7 shows the tensile properties of the GH4698 superalloy at RT and 700 °C after solution treatment at different temperatures followed by two-stage aging treatment, including ultimate tensile strength (UTS), yield strength (YS), and elongation. As the solution temperature increases, the strength of the alloy exhibits a decreasing trend at both test temperatures. Specifically, at RT, the UTS gradually decreases from 1268 MPa to 1226 MPa, and the YS decreases from 840 MPa to 807 MPa, while the elongation remains stable in the range of 28–32%. At 700 °C, the UTS decreases from 974 MPa to 904 MPa, and the YS decreases from 755 MPa to 696 MPa, with the elongation maintained at approximately 6%.

**Figure 8:**
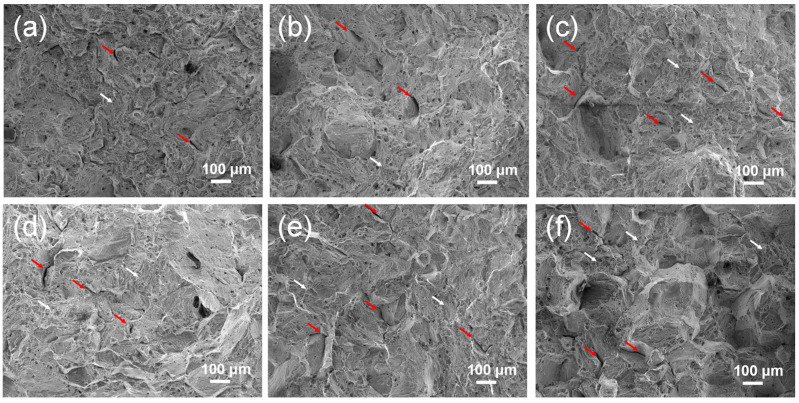
Tensile fracture characteristics of GH4698 superalloy at RT: (**a**) ST1060, (**b**) ST1080, (**c**) ST1090, (**d**) ST1100, (**e**) ST1120, (**f**) ST1150.

**Figure 9:**
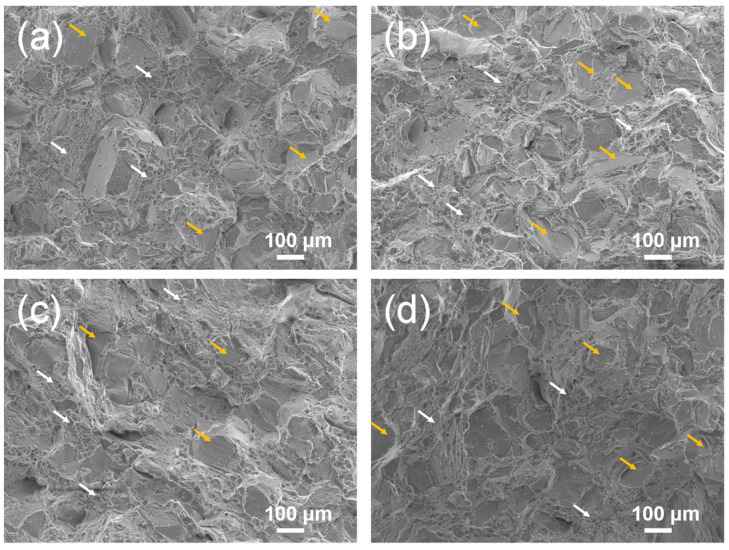
Tensile fracture characteristics of GH4698 superalloy at 700 °C: (**a**) ST1060, (**b**) ST1080, (**c**) ST1100, (**d**) ST1120.

Figure 8 shows the fracture morphology of the alloy after the tensile testing. As observed, all specimens exhibited typical ductile fracture characteristics at RT. The fracture surfaces were predominantly covered with numerous dimples (white arrows), along with a small number of intergranular cracks (red arrows). Moreover, fragmented blocky carbides could be observed within some dimples, indicating that carbides participated in the formation and coalescence of micro voids during deformation. At 700 °C, all specimens exhibited a mixed fracture mode, characterized by a composite morphology of dimples (white arrows) and cleavage facets (yellow arrows), as shown in Figure 9. The dimpled regions still retained certain ductile fracture features, although the dimples were shallower compared to those at RT. The appearance of cleavage facets, manifested as flat cleavage planes, confirmed the occurrence of brittle fracture. This mixed fracture morphology is consistent with the significant decrease in ductility of the alloy at elevated temperatures.

### 3.5. Contribution of Various Strengthening Mechanisms to YS at RT

As a typical Ni-based superalloy, the YS of GH4698 superalloy derives from the synergistic interaction between the multi-scale microstructure and multiple strengthening mechanisms. Among these mechanisms, the interaction between γ′ precipitates and dislocations constitutes the most fundamental strengthening contribution.

Moreover, the obstruction to dislocation motion by solute atoms in the matrix (solid solution strengthening) and the constraint on plastic deformation imposed by grain boundaries (grain boundary strengthening) also contribute significantly to the overall YS of the alloy. Generally, the total YS (σy) of the alloy can be approximately expressed as the linear superposition of the contributions from grain boundary strengthening (σgb), solid solution strengthening (σss), and precipitation strengthening (σp). The contributions of these strengthening mechanisms are represented as follows:

(1) Grain boundary strengthening. According to the Hall–Petch relationship, a high density of grain boundaries can effectively impede dislocation motion during tensile deformation. Grain coarsening reduces the grain boundary area, diminishing the contribution of grain boundary strengthening, which can be expressed as [27,28]:

(4) $$\sigma_{gb}=\sigma_{0}+k\cdot D^{-0.5}$$

Among these, *σ*_0_ represents the frictional stress, which is 37 MPa [29,30], and *k* is the Hall–Petch coefficient, which is determined to be 962 MPa/μm^0.5^ for the GH4698 superalloy, *D* is the average grain size, which can be obtained from Table 3.

(2) Solid Solution Strengthening. This strengthening mechanism arises from the long-range stress fields induced by elements such as Cr and Mo within the γ matrix. The variation in matrix composition under different solution temperatures can be determined as [31]:

(5) $$\sigma_{ss}=f_{\gamma }{\left[\sum k_{i}^{\frac{1}{p}}\cdot c_{i}\right]}^{p}$$

where *f*_γ_ is the volume fraction of the γ matrix, k_i_ and c_i_ are the solid solution strengthening constant and the concentration of solute atom *i* in γ matrix, respectively. The values of *k_i_* were selected based on the ref. [32], and *c_i_* was calculated using JMatPro 7.0 software, as shown in Table 5. *P* is a constant equal to 0.5.

**Figure 10:**
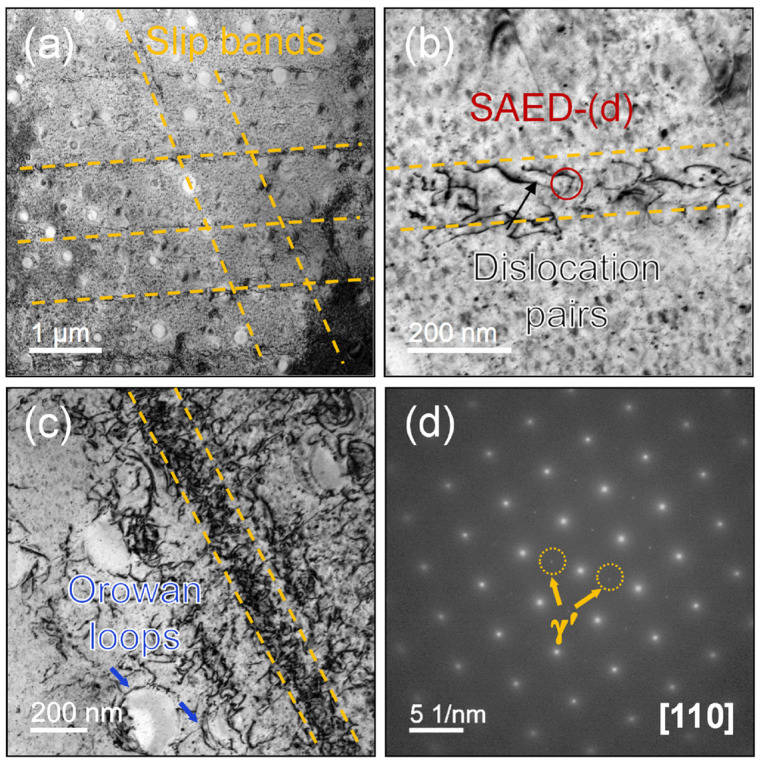
Dislocation configurations after tensile testing at RT: (**a**) Plane slip characteristics; (**b**) Dislocation pairs; (**c**) Curved dislocations and Orowan loops; (**d**) Corresponding SAED image in (**b**).

(3) Precipitation strengthening. Within the GH4698 superalloy, the γ′ precipitation phase is the critical factor impeding dislocation motion and enhancing strength. The effect of precipitation strengthening primarily depends on the interaction between the γ′ phase and dislocations and is dominated by the size of the precipitated phase. When the γ′ phase is small, dislocations tend to directly shear through the precipitation phase (shear mechanism). However, once the γ′ phase coarsens to the critical size, the critical resolved shear stress required for dislocation bypass becomes lower, leading to the dominance of the Orowan bypass mechanism [33,34]. Figure 10 shows the TEM analysis of the specimen after tensile fracture. As depicted in Figure 10a, the deformation characteristics primarily show planar slip, with multiple slip bands visible traversing the grains. A high density of dislocations is concentrated within the slip bands. The high magnification image in Figure 10b further reveals that these dislocations are arranged in the form of dislocation pair arrays. The selected area electron diffraction (SAED) pattern in Figure 10d shows distinct superlattice reflections from the γ′ phase, which, combined with the direct observation of dislocations shearing through the γ′ phase, confirms that during tensile deformation, dislocations primarily traverse the fine γ′ phases the shearing mechanism. According to the classical theory, when a full dislocation cuts into a γ′ precipitate with an L1_2_-ordered structure, it creates an anti-phase boundary (APB) within the precipitate, increasing significantly the system energy. To reduce the APB energy, this dislocation generally couples with another full dislocation of the same Burgers vector to form a dislocation pair, thereby restoring order in the sheared region of the γ′ phase. Meanwhile, around larger γ′ precipitates, no dislocation cutting is observed in Figure 10c; instead, dislocations are seen to pile up and bow around the precipitate interfaces, representing the typical Orowan bypass mechanism. These observations directly demonstrate that, due to the bimodal size distribution of γ′ phases in the GH4698 superalloy, both shearing and bypass mechanisms are activated during deformation. The synergistic interaction of these two mechanisms collectively enhances the overall strength and ductility.

Moreover, the γ′ phase shearing mechanism can be further classified into the “weak-pair coupling” and “strong-pair coupling” patterns based on the coupling strength [35,36]. When the γ′ phase radius is smaller than the critical value R_m_, the weak-pair mechanism is likely to occur. In this case, after the leading dislocation in a pair cuts through a γ′ phase, the trailing dislocation may not completely enter the same particle due to line-tension effects, leading to decoupling of the two dislocations between precipitates. In contrast, under strong-pair coupling conditions (γ′ radius larger than R_m_), the spacing between the two dislocations in a pair is comparable to the γ′ phase size, allowing the trailing dislocation to enter the same precipitate before the leading one fully exits, thereby maintaining a coupled state throughout [37]. The critical radius Rm that determines this transition in coupling behavior can be described theoretically by the following expression [38]:

(6) $$R_{m}=\frac{Gb^{2}}{2\gamma_{APB}}$$

where G is the shear modulus (87.4 GPa), *b* is the magnitude of the Burgers vector (0.254 nm), and *γ_APB_* is the APB energy of the γ′ phase on the {111} plane, taken as 0.2 J/m^2^. The calculated *R_m_* is 14.1 nm, which is smaller than the actual size of the fine γ′ phases in the present alloy (approximately 20 nm). Consequently, the shearing mechanism for the fine γ′ precipitates operates via strong-coupling dislocation shearing. Furthermore, the strengthening contribution of the fine γ′ particles can be expressed as [39]:

(7) $$\sigma_{scd}=\sqrt{\frac{3}{2}}M\left(\frac{Gb}{r}\right)\frac{f_{\gamma \prime_{s}}^{1/2}}{\pi^{3/2}}{\left(\frac{\pi d_{s}\gamma_{APB}}{Gb^{2}}-1\right)}^{1/2}$$

and the strengthening contribution of the large-size γ′ phases can be expressed as [36]:

(8) $$\sigma_{orowan}=\frac{3MGb}{{\left(\frac{2\pi }{3f_{\gamma \prime_{l}}}\right)}^{1/2}d_{l}}$$

Here, *M* is the Taylor factor (taken as 3.18), $f_{\gamma \prime_{s}}$ and $f_{\gamma \prime_{l}}$ are the volume fractions of the small and large γ′ phases, respectively, and *d_s_* and *d_l_* are the average diameters of the small and large γ′ phases, respectively. The total precipitation strengthening contribution is expressed as the linearly weighted sum of the strengthening contributions from the two sizes of γ′ phases.

**Figure 11:**
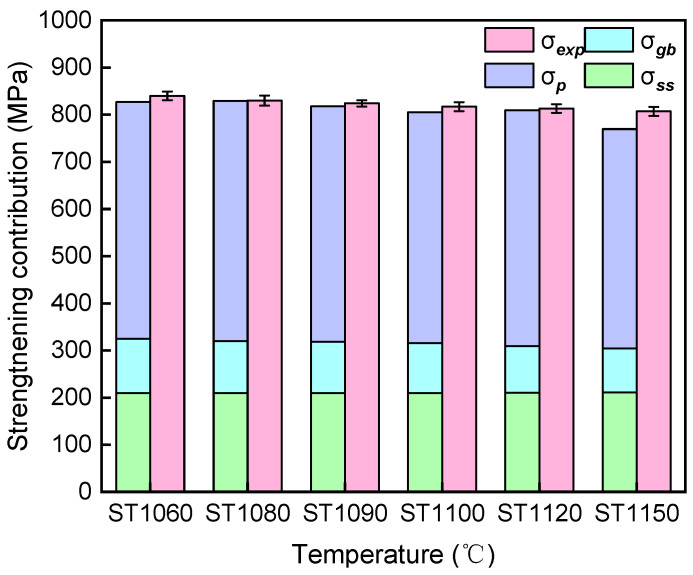
Comparison of the predicted YS and experimental values.

Figure 11 presents a comparison between the theoretically calculated and experimentally measured values of the YS, systematically analyzing the contributions of various strengthening mechanisms, including precipitation hardening, solid solution strengthening, and grain boundary strengthening. The analysis indicates that the YS is primarily attributed to the precipitation strengthening dominated by the γ′ phase, followed by the solid solution strengthening and grain boundary strengthening. The decrease in YS with increasing solution temperature is primarily due to the weakening of grain boundary strengthening caused by grain coarsening. Additionally, a certain discrepancy exists between theoretical calculations and experimental results. This deviation primarily stems from the simplified treatment of the deformation mechanism, particularly the insufficient consideration of additional strength increments induced by complex micro-deformation processes such as dislocation entanglement, which makes it difficult to fully reflect such effects in theoretical estimations.

### 3.6. Strength-Ductility Evolution Behavior at 700 °C

**Figure 12:**
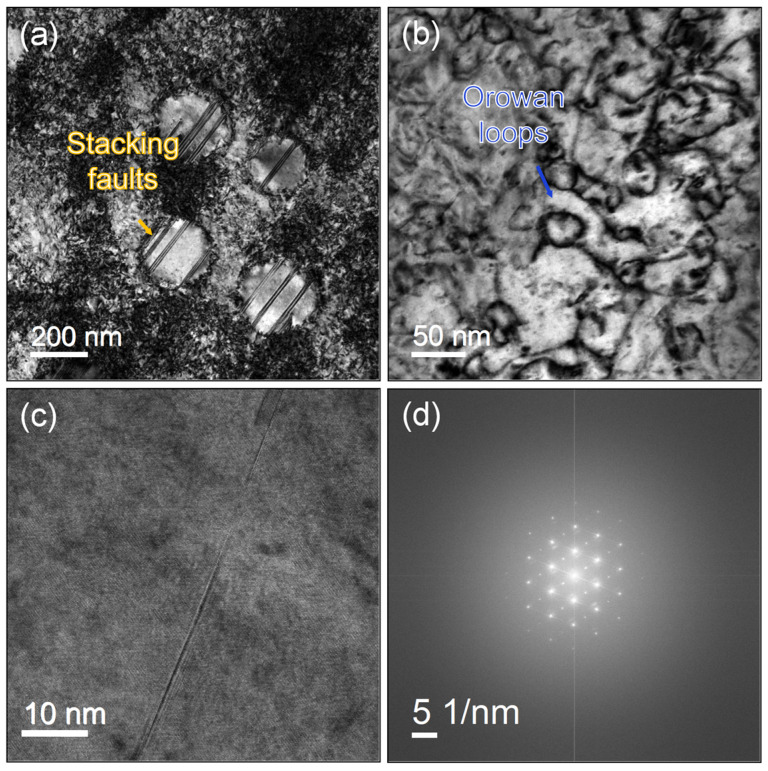
Dislocation configurations after tensile testing at 700 °C: (**a**) Stacking faults shearing through γ′ phases; (**b**) Curved dislocations and Orowan loops; (**c**) High-resolution image of stacking faults; (**d**) Corresponding SAED pattern of (**c**).

As discussed in Section 3.4, the strength of GH4698 alloy at 700 °C decreases with increasing solution treatment temperature. This trend is consistent with the room-temperature tensile properties, primarily due to reduced grain boundary strengthening resulting from grain coarsening. Moreover, compared to the performance at room temperature, the significant reduction in strength at 700 °C is closely associated with the transformation of deformation mechanisms at elevated temperatures. Figure 12 shows the microstructural characteristics after tensile deformation at 700 °C, where numerous stacking faults and Orowan dislocation loops are observed, indicating that stacking fault shearing dominates the high-temperature plastic deformation. In Ni-based superalloy, the interaction between dislocations and the γ′ phase directly influences the strengthening effect. At RT, dislocations primarily shear or bypass the γ′ precipitates through the dislocation pair shearing or Orowan mechanism, which requires a high critical resolved shear stress. However, under 700 °C, the thermal activation process promotes the dissociation of dislocations on {111} slip planes, forming extended dislocations and stacking faults [40]. When stacking faults shear the γ′ precipitates, the required critical resolved shear stress decreases significantly, leading to a reduction in the macroscopic strength of the alloy. The study by Zhang et al. [7] also confirms that the critical resolved shear stress required for stacking fault shearing is much lower than that for the Orowan bypass mechanism, which is an important microscopic mechanism responsible for the strength reduction at elevated temperatures. It is worth noting that the elongation at 700 °C is only about 6%, significantly lower than the 28–32% observed at RT, exhibiting a pronounced high-temperature embrittlement tendency. This phenomenon is primarily attributed to the combined effects of grain boundary weakening and carbide/matrix interface debonding at elevated temperatures.

## 4. Conclusions

This study systematically investigates the effects of solution temperature (1060–1150 °C) on grain growth kinetics, microstructural evolution, and tensile properties in GH4698 superalloy. The main conclusions are as follows:

(1) With increasing solution temperature, the Sellars grain growth kinetic model established based on experimental data indicates that the optimal time exponent is *n* = 3.4, with grain boundary migration activation energy *Q* = 478.7 kJ·mol^−1^.
(2) The quantitative statistical results indicate that the γ′ phase is independent of the solution temperature. However, the reduction in total grain boundary area caused by grain coarsening led to an increase in the number of M_23_C_6_ carbides per unit grain boundary length from 0.26 μm^−1^ to 0.39 μm^−1^.
(3) The tensile strength at room temperature decreases with increasing solution temperature, with the UTS dropping from 1268 MPa to 1226 MPa and the YS decreasing from 840 MPa to 807 MPa, while the elongation after fracture remains at 28–32%. At 700 °C, the UTS decreases from 974 MPa to 904 MPa, and the YS decreases from 755 MPa to 696 MPa, with the elongation maintained at approximately 6%. Quantitative analysis indicates that the reduction in strength is primarily attributed to grain coarsening.
(4) At 700 °C, the deformation mechanism transitions from dislocation shearing at room temperature to stacking fault shearing, resulting in a decrease in strength. In addition, grain boundary weakening leads to a reduction in elongation to approximately 6%.

## Figures and Tables

**Table 1:** The composition of GH4698 superalloy (wt.%).

Cr	Mo	Nb	Al + Ti	B	C	Ni
14.5	2.92	2.01	≥4	0.004	0.050	Bal.

**Table 2:** Designation of samples and heat treatment methods for tensile testing.

Name of Samples	Solution Treatment	Two-Stage Aging Treatment
ST1060	1060 °C × 8 h, air cooling	1000 °C × 4 h, air cooling + 775 °C × 16 h, air cooling
ST1080	1080 °C × 8 h, air cooling	
ST1090	1090 °C × 8 h, air cooling	
ST1100	1100 °C × 8 h, air cooling	
ST1120	1120 °C × 8 h, air cooling	
ST1150	1150 °C × 8 h, air cooling	

**Table 3:** Grain growth of GH4698 superalloy during solution treatment.

Temperature (°C)	Average Size (μm)			
	2 h	4 h	6 h	8 h
1060	52 (±6.56)	63 (±6.41)	72.4 (±4.64)	81.6 (±8.54)
1080	63 (±4.65)	78 (±5.97)	92 (±3.54)	105 (±4.32)
1090	74 (±3.54)	90 (±6.74)	101.2 (±6.58)	110 (±8.79)
1100	83 (±6.87)	96.5 (±8.54)	108.4 (±6.45)	120 (±8.42)
1120	96.7 (±6.45)	123 (±6.32)	131.8 (±8.76)	146.3 (±10.32)
1150	116.1 (±11.4)	140 (±13.2)	165 (±26.4)	175.3 (±15.64)

**Table 4:** Size, number density, and volume fraction of precipitated phases under different solution treatment temperatures.

Sample	Average Size of MC Carbide (μm)	Number Density of M_23_C_6_ Carbide (μm^−1^)	Volume Fraction of MC Carbide (%)	Volume Fraction of M_23_C_6_ Carbide (%)
ST1060	2.23 (±0.81)	0.26 (±0.14)	1.04 (±0.08)	0.25 (±0.02)
ST1080	2.14 (±0.52)	0.28 (±0.17)	1.08 (±0.03)	0.31 (±0.04)
ST1090	2.12 (±0.62)	0.31 (±0.21)	1.10 (±0.12)	0.28 (±0.02)
ST1100	2.21 (±0.47)	0.31 (±0.28)	1.12 (±0.16)	0.24 (±0.02)
ST1120	2.27 (±0.56)	0.34 (±0.21)	1.14 (±0.11)	0.27 (±0.01)
ST1150	2.02 (±0.71)	0.38 (±0.3)	1.08 (±0.12)	0.26 (±0.04)

**Table 5:** Detailed concentration distribution of γ matrix calculated by JMatPro software.

Sample	Cr	Mo	Al	Ti	Nb	Ni
ST1060	17.211	1.642	3.601	3.229	1.255	Bal.
ST1080	17.214	1.653	3.600	3.227	1.255	Bal.
ST1090	17.216	1.657	3.601	3.223	1.255	Bal.
ST1100	17.225	1.659	3.599	3.221	1.255	Bal.
ST1120	17.231	1.662	3.598	3.216	1.255	Bal.
ST1150	17.244	1.678	3.599	3.201	1.255	Bal.

## Data Availability

The original contributions presented in this study are included in the article. Further inquiries can be directed to the corresponding author.
